# In vivo confocal microscopy assessment of meibomian glands microstructure in patients with Graves’ orbitopathy

**DOI:** 10.1186/s12886-021-02024-z

**Published:** 2021-06-19

**Authors:** Shengnan Cheng, Yueqi Yu, Jin Chen, Lin Ye, Xinghua Wang, Fagang Jiang

**Affiliations:** 1 Department of Ophthalmology, Union Hospital, Tongji Medical College, Huazhong University of Science and Technology, Wuhan 430022, China; 2 Department of General Surgery, Union Hospital, Tongji Medical College, Huazhong University of Science and Technology, Wuhan 430022, China

**Keywords:** In vivo confocal microscopy, Meibomian glands, Microstructure, Graves’ orbitopathy

## Abstract

**Background:**

To evaluate microstructural changes in the meibomian glands (MGs) in patients with active and inactive Graves’ orbitopathy (GO), using in vivo confocal microscopy (IVCM), and to investigate the correlations between clinical and confocal findings.

**Methods:**

Forty patients (80 eyes) with GO (34 eyes with active GO, 46 eyes with inactive GO), and 31 age- and sex-matched control participants (62 eyes) were enrolled consecutively. A researcher recorded the clinical activity score (CAS) for each patient. A complete ophthalmic examination was then performed, including external eye, ocular surface and MGs. IVCM of the MGs was performed to determine the MG acinar density (MAD), MG longest and shortest diameters (MALD and MASD), MG orifice area (MOA), MG acinar irregularity (MAI), meibum secretion reflectivity (MSR), acinar wall inhomogeneity (AWI), acinar periglandular interstices inhomogeneity (API), and severity of MG fibrosis (MF).

**Results:**

All confocal microscopy assessments of MGs significantly differed among groups (all *P* = 0.000). Compared to controls, GO groups showed lower MOA (1985.82 ± 1325.30 μm^2^ in active GO and 2021.59 ± 1367.45 μm^2^ in inactive GO vs. 3896.63 ± 891.90 μm^2^ in controls, all *P* = 0.000) and MAD (87.21 ± 32.69 /mm^2^ in active GO and 80.72 ± 35.54 /mm^2^ in inactive GO vs. 114.69 ± 34.90 /mm^2^ in controls, *P* = 0.001 and 0.000, respectively); greater MALD (118.11 ± 30.23 μm in active GO and 120.58 ± 27.64 μm in inactive GO vs. 58.68 ± 20.28 μm in controls, all *P* = 0.000) and MASD (44.77 ± 19.16 μm in active GO and 46.02 ± 20.70 μm in inactive GO vs. 27.80 ± 9.90 μm in controls, all *P* = 0.000); and higher degrees of MAI, MSR, and MF (all *P*<0.05). Eyes with active GO had higher degrees of MAI (*P* = 0.015), AWI (*P* = 0.000), and API (*P* = 0.000), while eyes with inactive GO had higher degrees of MSR (*P* = 0.000) and MF (*P* = 0.017). In GO groups, AWI and API were positively correlated with CAS (*r* = 0.640, *P* = 0.000; *r* = 0.683, *P* = 0.000, respectively), and MF was negatively correlated with CAS (*r* = − 0.228, *P* = 0.042).

**Conclusions:**

IVCM effectively revealed microstructural changes of MGs in eyes with GO and provided strong in vivo evidence for the roles of obstruction and inflammation in the ocular surface disease process. Furthermore, it revealed discernible patterns of MG abnormalities in eyes with active GO and inactive GO, which are not easily distinguishable by typical clinical examinations.

**Supplementary Information:**

The online version contains supplementary material available at 10.1186/s12886-021-02024-z.

## Background

Graves’ orbitopathy (GO) is an inflammatory autoimmune disorder, that is associated with a combination of environmental, genetic, and immunological factors [1–3]. Ocular surface impairment is common in patients with GO, including conjunctival hyperemia and chemosis, dry eye, superficial punctate keratopathy, exposure keratopathy, and corneal ulcer [4–6]. Dry eye disease (DED) is the most common cause of ocular discomfort found in patients with GO [7]. It has been reported that up to 85% of patients exhibit dry eye [8, 9]. The pathogenesis of DED in patients with GO has not been completely elucidated. Some underlying mechanisms have been proposed, including mechanical factors [10–12], lacrimal gland impairment [13, 14], the changes of the composition and quantity of tears [15–17], the decrease of conjunctival epithelial cells and goblet cells, and the increase of inflammatory cells [18]. Patients with GO have dull corneal sensation, which leads to incomplete blinking, insufficient lacrimal gland secretion and unstable tear film. Corneal tear secretion is regulated by nerves; in patients with GO, the number of corneal nerve fibers decreases and their curvature increases, resulting in reduced tear secretion [5].

Dry eye is a multifactorial disease of the tears and ocular surface that results in symptoms of discomfort, visual disturbance, and tear film instability with potential damage to the ocular surface. It is accompanied by increased osmolarity of the tear film and inflammation of the ocular surface [19]. The International Dry Eye Work Shop classifies DED into aqueous tear deficiency and evaporative dry eye [20]. Meibomian gland dysfunction (MGD) is a chronic, diffuse abnormality of the meibomian glands, commonly characterized by terminal duct obstruction and/or qualitative/quantitative changes in the glandular secretion. This may result in alteration of the tear film, symptoms of eye irritation, clinically apparent inflammation, and ocular surface disease [21]. Inoue et al. [22] reported that eye discomfort and deterioration of the ocular surface in GO patients might be related to MGD. MGD is considered a major risk factor for evaporative dry eye [23]. MGD patients suffer from abnormalities of meibomian glands (MGs) in terms of both function and morphology. The anatomical features (i.e., the superficial location of MGs in the tarsal plates) allows MGs to be quantified via noncontact infrared meibography and in vivo confocal microscopy (IVCM). MG dropout refers to the loss of acinar tissue detected by meibography, and several investigators have shown that MG dropout is a useful index of obstructive MGD [24–26]. From a microscopy perspective, IVCM is reportedly useful for describing the morphological alterations of MGs in patients with MGD, such as reduction of MG acinar density, enlargement of acinar diameter, and contraction of MG orifices [27, 28]. IVCM has become an indispensable tool in the study of MGD, allowing direct noninvasive visualization of MG microstructures. Most previous studies focused on aqueous tear deficiency in patients with GO; however, they neglected MGD in these patients. Only a few studies have focused on MGs in patients with GO [22, 29, 30]. Thus, this study used IVCM to examine the morphological changes, particularly in terms of microstructure, in the MGs of patients with GO; it also evaluated correlations of these changes with clinical manifestations of GO.

## Methods

### Participants

This cross-sectional, observational study was approved by the Ethics Committee of Union Hospital affiliated to Tongji Medical College, Huazhong University of Science and Technology, Wuhan, China, and was conducted in strict accordance with the Declaration of Helsinki. All participants provided written informed consent prior to enrollment in the study. The sample size calculation was conducted prior to the enrollment by using PASS software version 15 (AQ5, NCSS, LLC, Kaysville, UT, USA), with 90% power, 0.05 alpha, and 0.50 effect size. After calculation, the minimum sample size was 78.

Patients diagnosed with GO were enrolled between March 2019 and January 2020 from our clinics. Patients with unilateral or bilateral GO were included. The diagnosis of GO was based on the Bartley criteria [31]. GO activity was classified on the basis of the clinical activity score (CAS) [32]. CAS ≥3/7 was considered indicative of active GO; CAS ≤2/7 was considered indicative of inactive GO [32]. Patients were excluded if they met either of the following criteria: presence of any other ophthalmic disorder or additional systemic disease or a history of previous eyelid or orbital surgery. Thirty-one age- and gender-matched patients without GO were enrolled as a control group, who were admitted to our hospital for cataract screening and met the same exclusion criteria.

### Clinical evaluation

All participants completed a patient history questionnaire including duration of GO; a researcher recorded the CAS for each patient. A complete ophthalmic examination was then performed. The external examination included proptosis, as well as assessments of palpebral fissure height (PFH) and lagophthalmos [33].

For all participants, the ocular surface status was evaluated by assessment of the following parameters: (1) Ocular surface disease index (OSDI), a valid and reliable instrument for measuring the severity of dry eye disease. According to the severity of dry eye symptoms, the OSDI score ranges from 0 to 100; asymptomatic patients are scored as 0 and the most symptomatic patients scored as 100. (2) Tear film lipid layer thickness (LLT) and partial blinking rate (PBR), detected via LipiView® Ocular Surface Interferometer (TearScience, Inc., Morrisville, NC, USA). An interferometric color unit (ICU) represented the lipid distribution; an ICU value of 1 indicated a tear film LLT of 1 nm [34, 35]. Because the exact LLT value cannot be measured or shown precisely by this instrument if the LLT value exceeds 100 nm, the LLT was artificially set to 100 nm for patients in whom LLT was ≥ 100 nm. (3) Noninvasive first breakup time (NIF-BUT), noninvasive average breakup time (NIAvg-BUT), and tear film breakup area (TBUA) [36]. A noninvasive method was used via Sirius (Sirius System CSO, Italian) to measure BUT to assess the stability of the tear film. The total duration of shooting was 17 s. (4) Schirmer I Test (SIT) [19, 36], used to evaluate the quantity of tears in each eye. The measurement was performed for 5 min without use of topical anesthesia. (5) Corneal fluorescein staining (CFS) [36], in which the cornea was divided into 4 quadrants and each quadrant was graded on a scale of 0 to 3. The total CFS of the 4 quadrants ranged from 0 to 12.

MG dropout was observed by means of noncontact infrared meibography (Sirius System, CSO) [37]; the ratio was automatically calculated within the system. Partial or complete loss of MGs was scored using the following grades for lower eyelid: 0, no loss of MGs; 1, area of loss less than one-third of the total MG area; 2, area of loss between one-third and two-thirds; 3, area of loss more than two-thirds [37]. To evaluate meibum quality, MGs in the central parts of the eyelid were assessed using a scale of 0–3: 0, clear fluid; 1, cloudy fluid; 2, cloudy particulate fluid; and 3, inspissated (similar to toothpaste). Five MGs in the central parts of the eyelid were evaluated to evaluate the MG expressibility on a scale of 0–3: 0, all glands expressible; 1, 3–4 glands expressible; 2, 1–2 glands expressible; and 3, no glands expressible [37].

### In vivo confocal microscopy

IVCM was performed on all subjects with a new-generation confocal microscope (HRT III Corneal Rostock Module, Heidelberg Engineering GmbH, Dossenheim, Germany) [38]. An experienced operator masked to the patients’characteristics performed the IVCM examinations. The MG acini and orifices were examined; corresponding images were collected and stored. Three non-overlapping, high-quality MG images were randomly selected from the nasal, central, and temporal sides of each lower eyelid (9 total images per eyelid) by 2 masked examiners and used for the analysis. The following variables were quantitatively determined [36, 39]: (1) MG orifice area (MOA; calculated automatically by ImageJ software, National Institutes of Health, Bethesda, MD, USA), (2) MG acinar density (MAD; acini were manually marked inside each 400*400-μm frame and the density was automatically calculated using the HRT III cell counting system), (3) MG acinar longest diameter (MALD), (4) MG acinar shortest diameter (MASD), (5) MG acinar irregularity (MAI) [36, 39], (6) meibum secretion reflectivity (MSR), (7) acinar wall inhomogeneity (AWI) [36, 39], (8) acinar periglandular interstices inhomogeneity (API), and (9) severity of MG fibrosis (MF) [36, 39]. The MAI was assessed on a 4-point scale, with virtually round or elliptical shape as 0, minimal presence of lobulated shaped acinar units as 1, moderate presence as 2 and heavy presence as 3 [40]. The MSR was evaluated on a 4-point scale [40–43]: black secretion color was scored as 0, dark gray color as 1, light gray color as 2, and white color as 3. The AWI and API were each rated on a 4-point scale: absence of punctate reflecting elements was scored as 0, minimal presence of punctuate reflecting elements as 1, moderate presence as 2, and heavy presence as 3 [40–43]. The MF was scored on a 3-point scale: no fibrosis was scored as 0, fibrosis in less than half of the lower eyelid as 1, and fibrosis in more than half of the lower eyelid as 2 [44].

### Statistical analysis

Data analysis was performed using IBM SPSS Statistics for Windows, version 20 (IBM Corp., Armonk, NY, USA). The normality of the data distribution was assessed by means of Shapiro-Wilk test. The inter-eye correlation was analyzed by using generalized estimating equations (GEE) models. For each variable, the Kruskal-Wallis test was used to ascertain statistical differences among the three groups. The Mann- Whitney U test, independent t-test, and X^2^ test were used to compare between pairs of groups when necessary. Bonferroni correction was applied for multiple comparisons. Spearman correlation coefficients were used to determine correlations of confocal microscopy data with clinical data in patients with GO. *P* < 0.05 was considered statistically significant.

## Results

### Clinical data

The demographic and clinical data in GO groups are shown in Table 1. We consecutively enrolled 40 patients with GO (80 eyes; 31 women and 9 men; mean age, 48.26 ± 10.41 years; range, 28–71 years), of which 34 eyes exhibited active GO and 46 eyes exhibited inactive GO. We also enrolled 31 control participants (62eyes; 21 women and 10 men; mean age, 45.65 ± 14.63 years; range, 18–73 years). Age and gender did not significantly differ among groups (both P>0.05).

**Table 1:** Clinical data of GO groups and controls

Clinical data	Active GO (34 eyes)	Inactive GO (46 eyes)	Controls (62 eyes)	P^*^
Age, years	50.65 ± 9.49	46.50 ± 10.80	45.65 ± 14.63	0.156^a^
Proptosis, mm	19.94 ± 3.83	17.63 ± 3.11	14.24 ± 1.06	0.000^b^
Palpebral fissure height, mm	11.37 ± 2.03	10.18 ± 1.78	8.76 ± 0.71	0.000^c^
Lagophthalmos, mm	0.99 ± 0.95	0.26 ± 0.65	0.00 ± 0.00	0.000^d^
CAS	3.56 ± 0.69	0.96 ± 0.72		0.000
Duration of GO, months	11.24 ± 12.18	16.82 ± 21.32		0.136
OSDI score	27.49 ± 15.11	23.14 ± 10.15	22.81 ± 18.93	0.345^e^
LLT, nm	73.09 ± 19.46	65.76 ± 13.68	66.98 ± 19.89	0.174^f^
PBR, %	51.74 ± 29.11	56.98 ± 25.03	37.10 ± 25.30	0.000^g^
NIF-BUT, s	6.09 ± 3.72	6.78 ± 3.86	8.20 ± 4.09	0.032^h^
NIAvg-BUT, s	8.26 ± 3.28	8.57 ± 3.61	9.58 ± 3.46	0.153^i^
TBUA, %	8.41 ± 9.65	8.16 ± 9.75	3.58 ± 5.17	0.005^j^
CFS	2.18 ± 2.15	0.72 ± 0.97	0.37 ± 0.55	0.000^k^
SIT, mm	7.00 ± 5.11	9.74 ± 4.00	10.77 ± 8.20	0.025^l^
MG dropout	1.62 ± 0.77	1.00 ± 0.29	0.82 ± 0.55	0.000^m^
Meibum quality	1.24 ± 0.60	1.02 ± 0.61	0.66 ± 0.65	0.000^n^
MG expressibility	1.09 ± 0.66	0.87 ± 0.74	0.71 ± 0.68	0.044^o^

Data are presented as mean ± standard deviation (SD), n (%) or median (range), as appropriate

*GO* Graves’ orbitopathy, *CAS* Clinical activity score, *OSDI* Ocular surface disease index, *LLT* Tear film lipid layer thickness, *PBR* Partial blinking rate, *NIF-BUT* Noninvasive first breakup time, *NIAvg-BUT* Noninvasive average breakup time, *TBUA* Tear film breakup area, *CFS* Corneal fluorescein staining, *SIT* Schirmer I test

* Obtained by Kruskal-Wallis test

^a^ Active GO vs. Inactive GO: *P* = 0.078; Active GO vs. Control: *P* = 0.206; Inactive GO vs. Control: *P* = 0.960 (Mann- Whitney U test)

^b^ Active GO vs. Inactive GO: *P* = 0.006; Active GO vs. Control: *P* = 0.000; Inactive GO vs. Control: *P* = 0.000 (Mann- Whitney U test)

^c^ Active GO vs. Inactive GO: *P* = 0.003; Active GO vs. Control: *P* = 0.000; Inactive GO vs. Control: *P* = 0.000 (Mann- Whitney U test)

^d^ Active GO vs. Inactive GO: *P* = 0.000; Active GO vs. Control: *P* = 0.000; Inactive GO vs. Control: *P* = 0.000 (Mann- Whitney U test)

^e^ Active GO vs. Inactive GO: *P* = 0.205; Active GO vs. Control: *P* = 0.056; Inactive GO vs. Control: *P* = 0.116 (Mann- Whitney U test)

^f^ Active GO vs. Inactive GO: *P* = 0.069; Active GO vs. Control: *P* = 0.116; Inactive GO vs. Control: *P* = 0.891 (Mann- Whitney U test)

^g^ Active GO vs. Inactive GO: *P* = 0.319; Active GO vs. Control: *P* = 0.019; Inactive GO vs. Control: *P* = 0.000 (Mann- Whitney U test)

^h^ Active GO vs. Inactive GO: *P* = 0.414; Active GO vs. Control: *P* = 0.013; Inactive GO vs. Control: *P* = 0.058 (Mann- Whitney U test)

^i^ Active GO vs. Inactive GO: *P* = 0.922; Active GO vs. Control: *P* = 0.135; Inactive GO vs. Control: *P* = 0.143 (Mann- Whitney U test)

^j^ Active GO vs. Inactive GO: *P* = 0.545; Active GO vs. Control: *P* = 0.000; Inactive GO vs. Control: *P* = 0.006 (Mann- Whitney U test)

^k^ Active GO vs. Inactive GO: *P* = 0.000; Active GO vs. Control: *P* = 0.000; Inactive GO vs. Control: *P* = 0.092 (Mann- Whitney U test)

^l^ Active GO vs. Inactive GO: *P* = 0.002; Active GO vs. Control: *P* = 0.037; Inactive GO vs. Control: *P* = 0.501 (Mann- Whitney U test)

^m^ Active GO vs. Inactive GO: *P* = 0.000; Active GO vs. Control: *P* = 0.000; Inactive GO vs. Control: *P* = 0.041 (Mann- Whitney U test)

^n^ Active GO vs. Inactive GO: *P* = 0.126; Active GO vs. Control: *P* = 0.000; Inactive GO vs. Control: *P* = 0.004 (Mann- Whitney U test)

^o^ Active GO vs. Inactive GO: *P* = 0.101; Active GO vs. Control: *P* = 0.011; Inactive GO vs. Control: *P* = 0.298 (Mann- Whitney U test)

**Fig. 1:**
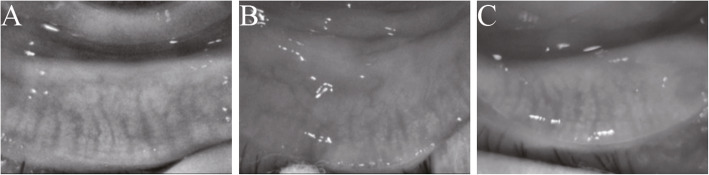
Infrared meibography images of meibomian gland (MG) dropout. Compared with controls (**A**), MG dropout was higher in active (**B**) and inactive Graves’ orbitopathy (**C**), which was most pronounced in active Graves’ orbitopathy (**B**)

All clinical parameters showed statistically significant differences among groups (all P<0.05), except duration of GO, OSDI, LLT and NIAvg-BUT (all P>0.05). PBR, NIF-BUT, TBUA and meibum quality in GO groups were significantly different from those parameters in controls (all P<0.05), but were not different between GO groups (all P>0.05). CFS and SIT in the active GO group were significantly different from those parameters in the inactive GO group and controls (all P<0.05), but were not different between the inactive GO group and controls (all P>0.05). MG expressibility was significantly different merely between the active GO group and controls (P<0.05). MG dropout was significantly greater in GO groups than in controls (all P<0.05), with values higher in the active GO group than in the inactive GO group (P<0.0001) (Fig. 1). In order to evaluate the inter-eye correlation, GEE analysis was performed which showed the same results. An Additional file shows this in more detail (see Additional file 1: Table S1).

### MG confocal data

**Fig. 2:**
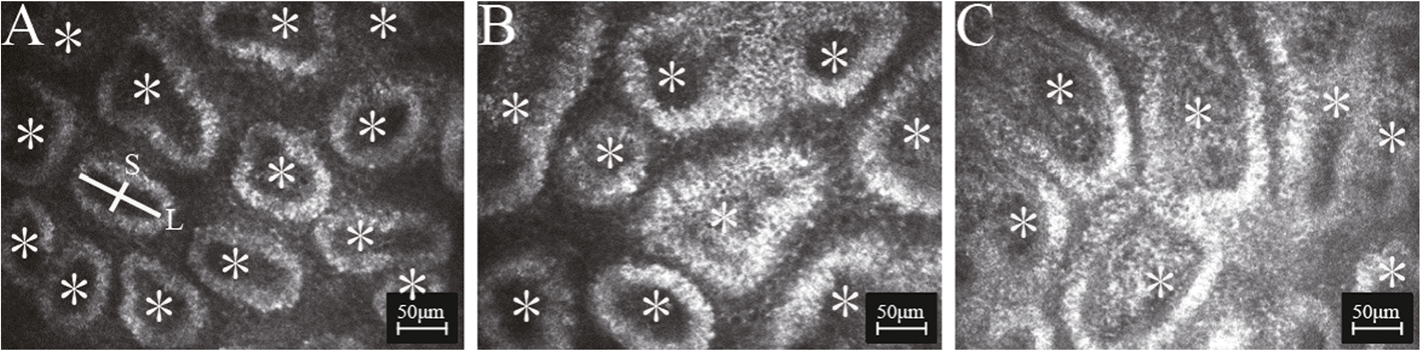
In vivo confocal microscopy images of meibomian gland (MG) acinar density (MAD), MG acinar longest diameter (MALD), and MG acinar shortest diameter (MASD). The multipoint tool was used to evaluate the number of clearly visible acinar unit (asterisk). The straight-line selection tool was used to trace and measure the MALD (L) and MASD (S). Compared with controls (**A**), the acini were obviously dilated and fused in active (**B**) and inactive Graves’ orbitopathy (**C**)

**Fig. 3:**
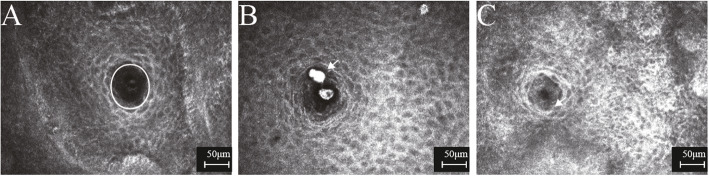
In vivo confocal microscopy images of meibomian gland orifice. The polygon tool was used to trace and measure meibomian gland orifice area (MOA). The orifice (highligted by a circle) was round, and the interior was uniform, presented low reflection in controls (**A**). However, the orifice was irregular in shape, with obvious blockage of high-reflective materials (arrow) in active Graves’ orbitopathy (**B**), and partial fibrosis (arrow) around the orifice in inactive Graves’ orbitopathy (**C**)

**Fig. 4:**
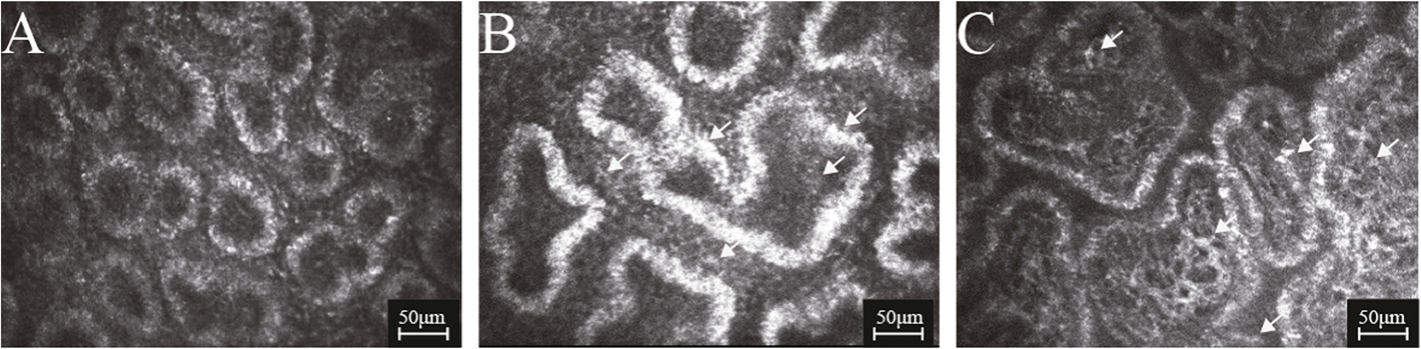
In vivo confocal microscopy images of meibomian gland acinar irregularity (MAI), meibum secretion reflectivity (MSR), acinar wall inhomogeneity (AWI), and acinar periglandular interstices inhomogeneity (API). The acini were oval, and arranged neatly, with connective tissues of interstice of the acini distributing homogeneously in controls (**A**). However, the acini were obviously irregular in shape and arranged disorderly in active (**B**) and inactive Graves’ orbitopathy (**C**). Particularly, the reflectivity of the acinar wall, as well as the intra- and extra-space of the acini were inhomogeneous in Graves’ orbitopathy (arrows)

**Fig. 5:**
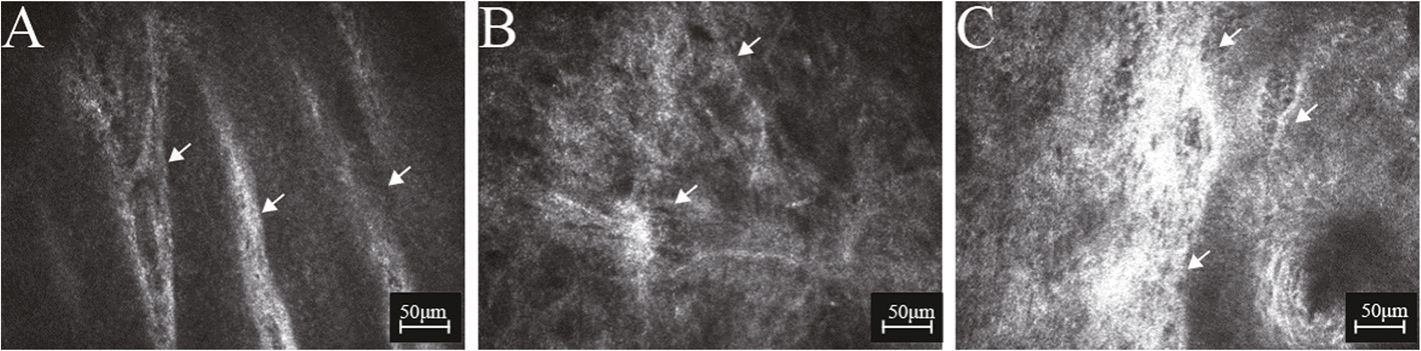
In vivo confocal microscopy images of meibomian gland fibrosis (MF). Compared with controls (**A**), excessive fibrosis (arrows) could be noted in active (**B**) and inactive Graves’ orbitopathy (**C**), which was most pronounced in inactive Graves’ orbitopathy (**C**)

All confocal microscopy assessments of MGs significantly differed among groups (all P<0.0001) (Table 2). MOA and MAD were significantly lower in active and inactive GO groups than in controls (all P<0.0001), and MALD and MASD were significantly greater in active and inactive GO groups than in controls (all P<0.0001); however, these four parameters did not differ between GO groups (all P>0.05) (Figs.2 and 3). AWI and API were significantly higher in the active GO group than in the inactive GO group and controls (all P<0.0001), but did not differ between the inactive GO group and controls (both P>0.05) (Fig. 4). MAI and MSR appeared significantly higher in the active and inactive GO groups, compared to controls (all P<0.05); MAI was more pronounced in the active GO group, while MSR was more pronounced in the inactive GO group (both P<0.05) (Fig. 4). MF was significantly greater in GO groups than in controls (both P<0.05), with values higher in the inactive GO group than in the active GO group (P<0.05) (Fig. 5). GEE analysis was performed to evaluate the inter-eye correlation which showed the same results. An Additional file shows this in more detail (see Additional file 2: Table S2).

**Table 2:** The confocal microscopy parameters of meibomian glands

Parameters	Active GO (34 eyes)	Inactive GO (46 eyes)	Control (62 eyes)	P^*^
MOA, μm^2^	1985.82 ± 1325.30	2021.59 ± 1367.45	3896.63 ± 891.90	0.000^a^
MAD, /mm^2^	87.21 ± 32.69	80.72 ± 35.54	114.69 ± 34.90	0.000^b^
MALD, μm	118.11 ± 30.23	120.58 ± 27.64	58.68 ± 20.28	0.000^c^
MASD, μm	44.77 ± 19.16	46.02 ± 20.70	27.80 ± 9.90	0.000^d^
MAI	1.59 ± 0.60	1.26 ± 0.49	0.71 ± 0.61	0.000^e^
MSR	1.21 ± 0.40	1.76 ± 0.73	0.94 ± 0.67	0.000^f^
AWI	1.94 ± 0.76	0.89 ± 0.60	0.97 ± 0.74	0.000^g^
API	2.06 ± 0.68	1.04 ± 0.62	0.90 ± 0.64	0.000^h^
MF	0.65 ± 0.59	0.93 ± 0.48	0.37 ± 0.55	0.000^i^

Data are presented as mean ± standard deviation (SD), n (%) or median (range), as appropriate

*MG* Meibomian gland, *MOA* MG orifice area, *MAD* MG acinar density, *MALD* MG acinar longest diameter, *MASD* MG acinar shortest diameter, *MAI* MG acinar irregularity, *MSR* Meibum secretion reflectivity, *AWI* Acinar wall inhomogeneity, *API* Acinar periglandular interstices inhomogeneity, *MF* Severity of MG fibrosis

^*^Obtained by Kruskal-Wallis test

^a^ Active GO vs. Inactive GO: *P* = 0.793; Active GO vs. Control: *P* = 0.000; Inactive GO vs. Control: *P* = 0.000 (Mann- Whitney U test)

^b^ Active GO vs. Inactive GO: *P* = 0.302; Active GO vs. Control: *P* = 0.001; Inactive GO vs. Control: *P* = 0.000 (Mann- Whitney U test)

^c^ Active GO vs. Inactive GO: *P* = 0.543; Active GO vs. Control: *P* = 0.000; Inactive GO vs. Control: *P* = 0.000 (Mann- Whitney U test)

^d^ Active GO vs. Inactive GO: *P* = 0.701; Active GO vs. Control: *P* = 0.000; Inactive GO vs. Control: *P* = 0.000 (Mann- Whitney U test)

^e^ Active GO vs. Inactive GO: *P* = 0.015; Active GO vs. Control: *P* = 0.000; Inactive GO vs. Control: *P* = 0.000 (Mann- Whitney U test)

^f^ Active GO vs. Inactive GO: *P* = 0.000; Active GO vs. Control: *P* = 0.040; Inactive GO vs. Control: *P* = 0.000 (Mann- Whitney U test)

^g^ Active GO vs. Inactive GO: *P* = 0.000; Active GO vs. Control: *P* = 0.000; Inactive GO vs. Control: *P* = 0.720 (Mann- Whitney U test)

^h^ Active GO vs. Inactive GO: *P* = 0.000; Active GO vs. Control: *P* = 0.000; Inactive GO vs. Control: *P* = 0.209 (Mann- Whitney U test)

^i^ Active GO vs. Inactive GO: *P* = 0.017; Active GO vs. Control: *P* = 0.020; Inactive GO vs. Control: *P* = 0.000 (Mann- Whitney U test)

### Correlations between clinical data and MG confocal data

Correlations between clinical data of patients with GO and MG confocal microscopy parameters were analyzed using Spearman correlation coefficients. The CAS score was negatively correlated with MSR (*r* = − 0.320, *P* = 0.004) and MF (*r* = − 0.228, *P* = 0.042), and was positively correlated with the degrees of MAI (*r* = 0.296, *P* = 0.008), AWI (*r* = 0.640, *P* = 0.000) and API (*r* = 0.683, *P* = 0.000). Proptosis (*r* = 0.272, *P* = 0.015), PFH (*r* = 0.233, *P* = 0.037), and lagophthalmos (*r* = 0.396, *P* = 0.000) were positively correlated with API; lagophthalmos was also positively correlated with AWI (*r* = 0.352, *P* = 0.001). The OSDI score was positively correlated with the degrees of MAI (*r* = 0.348, *P* = 0.002), AWI (*r* = 0.228, *P* = 0.042), and MF (*r* = 0.367, *P* = 0.001). The NIF-BUT was positively correlated with MSR (*r* = 0.267, *P* = 0.017), but was negatively correlated with AWI (*r* = − 0.234, *P* = 0.037). The CFS score was positively correlated with the degrees of MAI (*r* = 0.355, *P* = 0.001), AWI (*r* = 0.538, *P* = 0.000), and API (*r* = 0.503, *P* = 0.000). The SIT was positively correlated with MSR (*r* = 0.300, *P* = 0.007), but was negatively correlated with API (*r* = − 0.252, *P* = 0.024). MG dropout was positively correlated with the degrees of MAI (*r* = 0.505, *P* = 0.000), AWI (*r* = 0.630, *P* = 0.000), and API (*r* = 0.570, *P* = 0.000). Meibum quality and MG expressibility were positively correlated with the degrees of MAI (*r* = 0.497, *P* = 0.000; *r* = 0.473, *P* = 0.000), AWI (*r* = 0.327, *P* = 0.003; *r* = 0.466, *P* = 0.000), API (*r* = 0.380, *P* = 0.001; *r* = 0.320, *P* = 0.004), and MF (*r* = 0.282, *P* = 0.011; *r* = 0.427, *P* = 0.000).

## Discussion

In this study, we determined the microstructural changes of MGs in patients with GO, including lower MOA and MAD; greater MALD and MASD; and higher degrees of MAI, MSR, AWI, API, and MF, compared with age- and gender-matched healthy controls. Moreover, the active GO group had higher degrees of MAI, AWI, and API, as well as lower degrees of MSR and MF, compared with the inactive GO group. Most of our evidence suggests that an obstructive and inflammatory pathogenic mechanism contributes to morphological changes of MGs in patients with GO. To the best of our knowledge, our study is the first to use IVCM for analysis of differences in MG microstructure of patients with active and inactive GO.

IVCM has been recently applied to the armament of modalities used in the examination of MGs in patients with MGD, as well as many other ocular diseases (e.g., Sjogren’s syndrome [41], atopic keratoconjunctivitis [45] and primary blepharospasm [40]), providing a new noninvasive tool with which to study morphological changes in MGs. IVCM has been used by several researchers to investigate the effects of GO on the human cornea and bulbar conjunctiva at a cellular level [6, 18, 46]. Their observations suggested that GO induced changes in the bulbar conjunctiva, including reduced epithelial cell density and goblet cell density, increased Langerhans cell density, and higher degree of squamous metaplasia. GO also induced changes in the cornea, such as reduced corneal sensitivity, elevated numbers of corneal Langerhans cells, and abnormal corneal subbasal nerves.

Several causative mechanisms have been proposed to initiate the onset of dry eye in patients with GO, including MG abnormalities [22]. Kim et al. [47] suggested that absence of blinking in GO patients led to reduced secretion from MGs. This hypothesis was supported by the findings in a recent study by Park et al. [48], who observed that incomplete blinking and loss of MGs structure in the upper eyelid were more prominent in patients with GO than in dry eye patients. Moreover, CAS affected the structural loss of MGs in patients with GO. These results suggested that incomplete blinking and inflammation might be a major causative mechanism for MG changes in GO patients. In the present study, the structural loss of MGs (macrostructural MG dropout and microstructural MAD) was greater in GO groups than in controls; the active GO group had significantly greater loss of MGs than the inactive GO group. We postulated that this phenomenon can be attributed to the enhancement of incomplete blinking due to proptosis and palpebral fissure widening in patients with GO, with reduction of MG secretion and resultant obstruction. Our study showed that the PBR was significantly higher in GO groups, compared to controls; proptosis, PFH, and lagophthalmos were highest in the active GO group, which supports our hypothesis. Achtsidis et al. [49] reported that dry eye was common in early GO even in the absence of apparent proptosis; it was associated with CAS and reduced corneal sensitivity, which was consistent with our assumption that reduced corneal sensitivity in patients with GO could also lead to enhanced incomplete blinking, and MG dysfunction.

Several studies have shown strong associations between MG dysfunction and inflammatory ocular surface diseases [24, 50–52]. Villani et al. [5] found significant differences in the number of hyperreflective keratinocytes between eyes with active and inactive GO by using corneal confocal microscopy. Previous researchers reported that tear concentrations of cytokines IL-1β, IL-2, IL-17A, IL-6, IL-7, IL-10, and TNF-α were significantly higher in patients with GO; moreover, there were significantly positive correlations between some of the indicator levels and CAS [15–17]. Yoon et al. [53] demonstrated tear nerve growth factor concentrations might have a specific role in ocular surface inflammation, which protected against ocular surface damage in patients with active GO. Anti-inflammatory treatment significantly reduced the nerve growth factor level in tears, increased tear film stability and production, and diminished congestive symptoms. Wang et al. [30] also described functional and morphological MG alterations in patients with GO. Notably, they found that patients with active GO had more severe MG dropout. They assumed that active GO may cause periglandular MG inflammation, leading to MG dropout. These results indicated that orbital inflammation may be involved in ocular surface damage in patients with GO. Thus, GO-associated ocular surface inflammation might cause periglandular inflammation and subsequent MG dysfunction.

Previous studies reported that patients with obstructive MGD showed comparative morphological changes including MG loss, greater acinar diameters, higher secretion reflectivity, and greater AWI and API [27, 41, 45]. These alterations may be due to qualitative changes in MG secretion (inspissation, increased viscosity, and build-up) and resultant MG obstruction. Thus, the MGs of patients with MGD and those with GO differ only in terms of MG orifice size. We hypothesized that the reduced orifice area in patients with GO might involve inflammatory-mediated fibrosis and duct atrophy, with difficulty meibum secretion and resultant orifice obstruction. The gland productivity continues, but meibum cannot be secreted and the lipid pool storage increases. This leads to acinar unit dilation and enhanced values of MALD and MASD.

The MAI and MSR significantly increased in both active and inactive GO groups; however, the degree of MAI was more pronounced in active the GO group, while the degree of MSR was more pronounced in the inactive GO group. These alterations might be translated to glandular structural disorder and enhanced lipid pool storage, respectively. In our study, the duration of GO was longer in the inactive GO group (16.82 ± 21.32 months) than in the active GO group (11.24 ± 12.18 months). The natural history of GO is characterized by an active inflammatory phase of approximately 1–3 years, followed by an inactive fibrotic phase; thus, combined with the finding that the degree of MF was greater in the inactive GO group, we inferred that MG fibrosis resulted in lipid deposition in acini, which ultimately led to the most pronounced MSR in eyes with inactive GO. Concurrently, inflammation-mediated irregularities in acinar structures were more prominent in eyes with active GO. This aspect was supported by the positive correlation between MAI and CAS, and the negative correlation between MSR and CAS.

In our study, the AWI and API also significantly increased in the active GO group, compared to the inactive GO group and controls. As suggested by Villani et al. [41, 42] for other conditions, the enhanced inhomogeneity may constitute a sign of inflammation in the tarsus and MGs. However, Osama et al. [45] found confocal microscopy evidence of MG fibrosis in patients with atopic keratoconjunctivitis, which may led to the enhanced API. Our study did not reveal direct evidence of inflammation (e.g., dendritic cells) in eyes with GO. Previous studies demonstrated that quantitative dendritic cell density could reveal periglandular inflammation [27, 38]. In our study, the typical dendritic shape was often unrecognizable and individual cells could not be clearly identified by using IVCM. Thus, we chose to perform a semiquantitative evaluation of punctiform elements visible in the interstices. Furthermore, we found strong positive correlations of both AWI and API with CAS. Therefore, we regarded the AWI and API as confocal microscopy signs of eyelid margin and tarsal inflammation. In this study, we also investigated the confocal microscopy signs of the degree of MG fibrosis, which was significantly greater in the active and inactive GO groups, compared to controls. We suspect that MG fibrosis is exacerbated by inflammation involving interactions between inflammatory cells and fibroblasts; subsequent activation leads to pathogenic fibrosis in the MG microenvironment.

We found that eyes with GO were characterized by greater MG dropout, tear film instability (shorter BUT), and ocular surface epithelial damage (higher CFS), as well as worser MG secretion; some of these findings were consistent with previous reports [30, 47, 48]. In our study, the SIT between the inactive GO group and controls showed no significant difference. However, in patients with active GO, tear secretion was significantly reduced, compared to the inactive GO group and controls. These results were consistent with some previous studies [13, 48], although others demonstrated conflicting findings [30, 54]. Eckstein et al. [13] reported that high TSHR autoantibody activity in patients with active GO might impair the lacrimal gland, which could physiologically express TSHR. Notably, we found no significant differences in LLT among the active GO group, inactive GO group, and controls; this finding was parallel with the results of Park et al. [48], but abhorrent to the results of Wang et al. [4] As suggested by Park et al., we also consider that the number of blinks must be compared to investigate LLT in the active and inactive GO groups. Otherwise, it was not convincing to explain why a thicker LLT but higher MGD in eyes with active GO, solely on the basis of lagophthalmos-mediated forceful blinking.

There were some limitations in this study. First, we only performed confocal microscopy analysis of the inferior eyelid margin. Confocal examination requires a few minutes of contact between the instrument and examined tissue, which can be uncomfortable for the subject. Moreover, the examination involves a fixed orientation, in which the examined tissue must be positioned parallel to the face of the sterile cap. The inferior eyelid margin provides a less invasive approach for the visualization of glandular structures. Second, the control group is not representative of the healthy population. Some participants in the control group also had dry eye symptoms and inadequate MG performance, but these characteristics did not appear to bias our findings. Third, we designated LLT as 100 nm for eyes with LLT > 100 nm, which may have caused underestimation of the average LLT value. The exact LLT value cannot be measured precisely or shown by this instrument if it exceeds 100 nm, which is the limit of the measuring instrument; we cannot improve this measurement without a more accurate measuring instrument.

## Conclusions

IVCM offers a new approach for in vivo noninvasive histopathological evaluations of MGs in eyes with GO. Our study revealed new evidence regarding the pathologic morphological changes of MGs in eyes with GO and provided strong in vivo evidence for the roles of obstruction and inflammation in the ocular surface disease process. These findings aid in elucidating mechanism underlying GO-associated dry eye. Moreover, these findings showed that active and inactive GO can be identified by their discernible patterns of MG abnormalities, whereas they cannot be easily distinguished by typical clinical examinations. In patients with active GO, we commonly observed confocal microscopy signs of inflammation and slight dilatative morphological changes; in patients with inactive GO, we commonly observed signs of glandular obstruction, distension, and fibrosis.

## Supplementary Information

**Additional file 1: Table S1.** Comparison of clinical data among active GO group, inactive GO group, and controls.

**Additional file 2: Table S2.** Comparison of confocal microscopy parameters of meibomian glands among active GO group, inactive GO group, and controls.

## Data Availability

The datasets used and analyzed during the current study are available from the corresponding authors on reasonable request.

## Abbreviations

GO: Graves’ orbitopathy
CAS: Clinical activity score
OSDI: Ocular surface disease index
LLT: Tear film lipid layer thickness
PBR: Partial blinking rate
NIF-BUT: Noninvasive first breakup time
NIAvg-BUT: Noninvasive average breakup time
TBUA: Tear film breakup area
CFS: Corneal fluorescein staining
SIT: Schirmer I test
MG: Meibomian gland
MOA: MG orifice area
MAD: MG acinar density
MALD: MG acinar longest diameter
MASD: MG acinar shortest diameter
MAI: MG acinar irregularity
MSR: Meibum secretion reflectivity
AWI: Acinar wall inhomogeneity
API: Acinar periglandular interstices inhomogeneity
MF: Severity of MG fibrosis.
