# Supplementary material for: In vivo confocal microscopy assessment of meibomian glands microstructure in patients with Graves’ orbitopathy
Source: BMC Ophthalmol. 2021 Jun 19;21:261. doi: 10.1186/s12886-021-02024-z (PMC8214770; doi:10.1186/s12886-021-02024-z)
Supplement: Supplementary file 1 — Additional file 1: Table S1. Comparison of clinical data among active GO group, inactive GO group, and controls. [file 12886_2021_2024_MOESM1_ESM.docx]

Table S1 Comparison of clinical data among active GO group, inactive GO group, and controls

| Clinical data | OR (95 % CI) | P |
| --- | --- | --- |
| Age，years | 0.997 (0.994, 1.001) | 0.141 |
| Proptosis, mm | 0.050 (0.023, 0.110) | 0.000 |
| Palpebral fissure height, mm | 0.278 (0.184, 0.421) | 0.000 |
| Lagophthalmos, mm | 0.613 (0.507, 0.741) | 0.000 |
| CAS | 0.083 (0.059, 0.116) | 0.000 |
| Duration of GO, months | 1.323 (0.657, 2.665) | 0.433 |
| OSDI score | 0.998 (0.994, 1.003) | 0.406 |
| LLT, nm | 0.051 (0.000, 9.241) | 0.262 |
| PBR, % | 0.906 (0.842,0.975) | 0.008 |
| NIF-BUT, s | 2.926 (1.269, 6.749) | 0.012 |
| NIAvg-BUT, s | 1.984 (0.962, 4.095) | 0.064 |
| TBUA, % | 0.096 (0.013, 0.723) | 0.023 |
| CFS | 0.370 (0.230, 0.595) | 0.000 |
| SIT, mm | 1.523 (1.247, 1.949) | 0.026 |
| MG dropout | 0.697 (0.589, 0.823) | 0.000 |
| Meibum quality | 0.709 (0.622, 0.808) | 0.000 |
| MG expressibility | 0.776 (0.706, 0.872) | 0.033 |

GO, Graves’ orbitopathy; OR, odd ratio; CI, confidence interval; CAS, clinical activity score; OSDI, ocular surface disease index; LLT,tear film lipid layer thickness; PBR, partial blinking rate; NIF-BUT, noninvasive first breakup time; NIAvg-BUT, noninvasive average breakup time; TBUA, tear film breakup area; CFS, corneal fluorescein staining; SIT, Schirmer I test; MG, meibomian gland.

P values were based on repeated-measures modeling using generalized estimating equations (GEE).

All clinical parameters showed statistically significant differences among three groups (all P＜0.05), except duration of GO, OSDI, LLT and NIAvg-BUT (all P＞0.05).
