# Supplementary material for: In vivo confocal microscopy assessment of meibomian glands microstructure in patients with Graves’ orbitopathy
Source: BMC Ophthalmol. 2021 Jun 19;21:261. doi: 10.1186/s12886-021-02024-z (PMC8214770; doi:10.1186/s12886-021-02024-z)
Supplement: Supplementary file 2 — Additional file 2: Table S2. Comparison of confocal microscopy parameters of meibomian glands among active GO group, inactive GO group, and controls. [file 12886_2021_2024_MOESM2_ESM.docx]

Table S2 Comparison of confocal microscopy parameters of meibomian glands among active GO group, inactive GO group, and controls

| Parameters | OR (95 % CI) | P |
| --- | --- | --- |
| MOA, μm2 | 2.701 (2.005, 3.637) | 0.000 |
| MAD, /mm^2^ | 1.161 (1.069, 1.260) | 0.000 |
| MALD, μm | 0.969 (0.963, 0.975) | 0.000 |
| MASD, μm | 0.991 (0.987, 0.994) | 0.000 |
| MAI | 0.685 (0.589, 0.796) | 0.000 |
| MSR | 0.815 (0.696, 0.955) | 0.011 |
| AWI | 0.608 (0.505, 0.733) | 0.000 |
| API | 0.566 (0.479, 0.670) | 0.000 |
| MF | 0.835 (0.719, 0.970) | 0.018 |

OR, odd ratio; CI, confidence interval; MG, meibomian gland; MOA, MG orifice area; MAD, MG acinar density; MALD, MG acinar longest diameter; MASD, MG acinar shortest diameter; MAI, MG acinar irregularity; MSR, meibum secretion reflectivity; AWI, inhomogeneous appearance of walls of acinar units; API, inhomogeneous appearance of periglandular interstices of acinar units; MF, severity of MG fibrosis.

P values are based on repeated-measures modeling using generalized estimating equations (GEE).

All confocal microscopy assessments of Meibomian glands significantly differed among three groups (all P＜0.05).
